# Effectiveness of a resilience, gender equity and mental health group intervention for young people living in informal urban communities in North India: a cluster randomized controlled trial

**DOI:** 10.1080/16549716.2025.2455236

**Published:** 2025-02-03

**Authors:** Varadharajan Srinivasan, Miguel San Sebastián, Samson Rana, Pooja Bhatt, Greg Armstrong, Smita Deshpande, Kaaren Mathias

**Affiliations:** a Project Burans, Herbertpur Christian Hospital (Emmanuel Hospital Association), New Delhi, India; b The George Institute for Global Health, Jasola Vihar, New Delhi, India; c Department of Epidemiology and Global Health, Umeå University, Umeå, Sweden; d Melbourne School of Population & Global Health, The University of Melbourne, Victoria, Australia; e Centre of Excellence in Mental Health, ABVIMS - Dr RML Hospital, New Delhi, India; f Faculty of Health, University of Otago, Dunedin, New Zealand

**Keywords:** Youth, mental, intervention, resilience, India

## Abstract

**Background:**

Mental health problems are the leading cause of disease burden among young people in India. While evidence shows that youth mental health and resilience can be improved with group interventions in school settings, such an intervention has not been robustly evaluated in informal urban settings.

**Objective:**

This study aimed to evaluate whether the Nae Disha 3 group intervention could improve youth resilience, mental health and gender equal attitudes among disadvantaged young people from low-income urban communities in India.

**Methods:**

This cluster randomised controlled trial used an analytic sample of 476 adolescents and young adults aged 11–25 years from randomised clusters in urban Dehradun, India. The 251 intervention group participants were 112 boys and 139 girls, and the 225 young people in the wait-control group were 101 boys and 124 girls. Five validated tools measuring resilience gender equity and mental health were filled by participants at three different points in time.

**Results:**

Difference in difference (DiD) analysis at T2 showed that scores improved among girls in intervention group, for adjusted model, resilience (DiD = 4.12; 95% CI: 2.14, 6.09) and among boys, for resilience (DiD = 5.82; 95% CI: 1.57, 9.74).

**Conclusions:**

The Nae Disha 3 intervention among disadvantaged urban youth moderately improved resilience for both young men and women, though it did not significantly impact mental health, self-efficacy, or gender-equal attitudes. We establish potential merit for this approach to youth mental health but recommend further research to examine active ingredients and the ideal duration of such group interventions.

## Paper context

- **Main findings:** This low-cost resilience, gender equality and mental health intervention led to improved resilience for both young women and men but did not show a statistically significant improvement in gender equal attitudes or mental health.
- **Added knowledge:** This intervention, implemented in a low-income urban setting, was feasible and acceptable, particularly for young women, and effective at strengthening resilience for both young men and women.
- **Global health impact for policy and action:** A community-based group intervention for disadvantaged young people in urban North India was moderately effective and feasible, yet further research is required to further examine implementation and effectiveness.

## Background

One in five young people globally currently experiences mental health problems and mental ill-health accounts for 45% of the burden of disease for people aged 10–24 years [1,2]. Poor mental health limits the health, survival and the future for young people globally by reducing access to education, employment and social networks which all negatively impact their life course as adults [3]. India has 600 million young people, more than any other country in the world [4] and for youth, depression is one of the leading causes of years lived with disability [4]. A recent meta-analysis showed a pooled prevalence of depression of 27% in rural Indian adolescents [5,6].

Psychosocial resilience is the ability to function during or after adversity [7] and is a developmental construct that can explain ways that people can thrive. Psychosocial resilience can increase a young person’s mental and physical health and also provide a way to engage with challenges over time [8]. Evidence suggests that adolescents participating in interventions targeted at improving psychosocial interventions show improved performance in school and mental health in early adulthood [9]. A number of psychosocial adolescent youth and resilience interventions profiled in a recent scoping review showed promising improvements in mental health and resilience [10,11]. However, review authors identified the significant research gap in examining the effectiveness of such interventions among disadvantaged and out-of-school young people [11]. A key group among these are youth who reside in informal urban communities who experience many stressors such as poverty, parents with limited education, poor quality housing, family violence, low access to all healthcare and high rates of school drop-out [12,13]. A recent study underlined the interconnectedness of social health determinants and poor mental health, particularly in informal urban settings in India [13]. A further review has also underlined this research gap and described the urgent need for intervention studies targeting marginalised young people, such as those residing in informal urban communities [14].

Gender is a significant social determinant of health, particularly in India which is ranked 140th in the Global gender gap report [15], Other indicators of the hegemonic masculinity in India include low sex ratios at birth (991 females to 1000 males) [16] and the large gap in literacy between women and men. The patriarchy also combines with age hierarchy so that decisions about education, marriage and career choices of young people are often made by older relatives, which contributes to poor youth mental health increasing the risks for common mental disorders indicating a tight triad linking psychosocial distress, gender disadvantage and poor health outcomes [17]. A recent study among young women in a low-income city in India found higher levels of gender disadvantage correlated with poorer resilience and greater psychological distress, suggesting these are connected as three sides of a triangle [18]. However, gender attitudes can change through participation in targeted interventions, although parental support continues to operate as an enabler [18,19]. Gender transformative interventions in India have been shown to improve health outcomes and reduce school dropouts [9,20,21]. Recent studies therefore point to the merits of broad youth development interventions which include financial literacy, sexual and reproductive health components and also show the need for further investigation into community-based interventions that seek to increase gender equity [20,22,23].

Nae Disha is an 18-week community-based psycho-social, gender-transformative, manualised, group intervention developed by a group which included public health physicians, social workers, young people and youth workers, who developed the 15 interactive sessions seeking to promote youth resilience, gender equal attitudes and mental health. It was iteratively developed using youth development and positive psychology strengths-based perspectives to build external assets such as support, empowerment, boundaries and expectations and internal assets such as commitment to learning and positive identity [24]. Nae Disha 3 (ND3) was the product of further revision of the intervention in 2018 to improve its relevance for young men, to include a wider age range and modules around sexual and reproductive health [23] Earlier versions (ND1 and ND2) among disadvantaged young women and among young people impacted by psycho-social disability were evaluated with pre-post quantitative studies, and in both instances achieved significant improvements in validated psychometric measures of mental health, resilience [25] and social inclusion [26,27]. Our realist evaluation of Nae Disha identified mechanisms that triggered outcomes included forming new peer friendships, perceiving safe spaces for speaking out, and taking collective action [27], findings noted elsewhere [28]. However, none of these evaluations used a controlled design.

The aim of this study was to assess the effectiveness of the ND3 intervention on youth resilience, mental health and gender equal attitudes among young people in the North Indian state of Uttarakhand using a randomised controlled design. We expected resilience, positive mental health and gender equal attitudes to improve in the intervention groups over the wait-control group between pre-test and post-test measures.

## Methods

### Study design and sample size calculations

**Figure 1.**
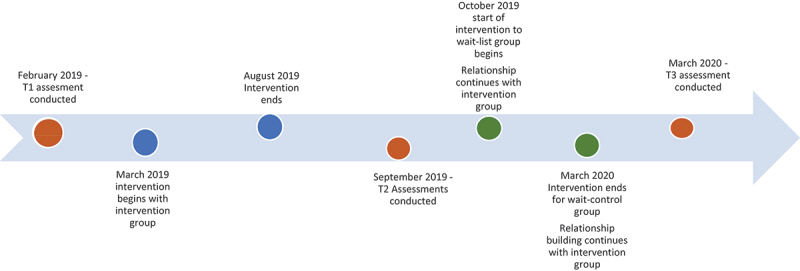
Timeline showing time vs intervention and assessment components for intervention and wait-control groups.

The study used a cluster randomised controlled trial, with randomising at the cluster level. The intervention was first provided to the participants in the intervention group, and this was followed by pre-post (uncontrolled) methodology with the wait-control group receiving the intervention in the interval between the post-intervention and follow-up data collection in the intervention group (Figure 1). This pragmatic design was applied to meet ethical requirements to complete the intervention with both intervention and control groups and funding requirements to complete the study within 12 months. The study was approved as protocol 192 by the Institutional Ethics Committee of Emmanuel Hospital Association, New Delhi. The study is registered as trial CTRI/019/02/017614 in the Clinical Trial Registry, India.

**Figure 2.**
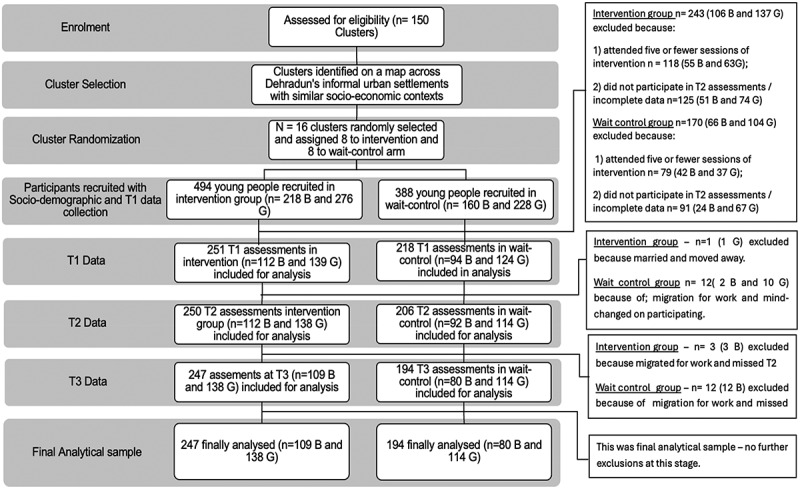
The consolidated standards of reporting trials (CONSORT) diagram information is presented in order to identify any differential dropout between the arms of the Nae Disha trial.

Sample size was calculated using optimal design software with these assumptions: 1) intra-cluster correlation of 0.05 based on the CYRM outcome intraclass correlation, with a power of 80% and alpha level of 0.05. We used a standardized design effect size of 1 yielding a sample size of 20 participants per cluster in eight clusters. With an estimated 20% attrition, the final sample size was calculated to be 160 boys and girls each in eight clusters in the intervention arm and 160 boys and girls each in eight clusters in the control arm (detailed time line of the interventions and assessments can be found in Figures 1 and 2) assumptions were built on published trials of this intervention that showed high attendance and highly significant outcomes for participants [29,30].

### Cluster selection and recruitment

Young people participating in the study, resided in informal urban communities (slums) in Dehradun district, Uttarakhand. These settlements typically are densely crowded and lack potable water, garbage disposal, health services and sanitation facilities [31] and slum residents often have poor health and educational indicators. We used a map to identify 150 clusters from within the 12 slums we had identified, and these were numbered 1 to 150. Each cluster was spaced at a minimum of 200 m from another cluster, which we believed would be sufficient to limit mixing between intervention and wait-control group participants. This assumption was built on our experience working with young women who had very limited freedom of movement and were often not acquainted with peers living just 20 m away. Using a digital random generator with a range of 1 to 150, we identified eight clusters where participants were recruited from. Each cluster included one group of girls and boys (*n* = 10 in each), and the clusters chosen for control and interventions were ensured to be far enough so that young people recruited for control groups, were not in contact with the intervention groups between time periods T1 and T2. The control group participants were made aware that they would be provided the intervention after 6 months; however, they were not aware that the intervention was to be provided for the intervention group immediately. The consents were sought from parents, if the participants were below 18 years of age.

### Participant details

The initial recruitment with sociodemographic data included 882 young adults for both arms. Data was collected from these participants at T1, T2 and T3; however, T1 data from only 469 participants were included for analysis. The data from participants with attendance at five or fewer sessions or incomplete data were excluded. Twenty-eight of these young people were not available when we conducted follow-up measures, rendering a final sample who completed all three measures of 189 boys and 252 girls (total of 441 young people). In the case of boys, there were 109 in the intervention group and 80 in the wait-control group, while in the case of girls, 138 were in the intervention group and 114 in the wait-control group.

The lower numbers of participants in the wait-group were fewer recruited (not wanting to sign up if the intervention was to be delayed) and also loss to follow up for young men who were not available during the intervention or for T2 and T3 due to income generation opportunities (weddings and harvesting). The young men participating had an older mean age due to gender norms assigning household responsibilities to young women over 16 years making younger women more available.

## Intervention

The intervention had two formal components: The first relationship building component occurred for 2 weeks prior to starting the intervention meaning February 2019 to March 2020 for the intervention group and from October 2019 to March 2020 for the control group (Figure 1). Facilitators sought to respond to the concerns of youth and their families. For example, mothers described concerns about their youth playing games out of their community, so facilitators moved the group meeting location so that youth could meet within their geographical community.

The second component facilitated group meetings of an 18-module curriculum (Nae Disha 3 (ND3)). Group meetings lasted 50–60 min and included games, physical movement, role-plays and interactive approaches and an at-home activity each week. The intervention was developed for young people aged 14–19 years but was adapted to meet the needs of younger or older youth by facilitators. The 18 modules are summarised in Table 1. In the months post-intervention, young people were provided with access to a community meeting room where youth continued to meet together informally to play games or talk together, and they also organised sports events and a youth day celebration, which provided opportunities for leadership. The entire module is available for open access download at https://burans.org/wp-content/uploads/2020/12/nae-disha.pdf.

**Table 1.** Outline of structured components of nae disha 3.

Theme	Modules	Sample of content
*Self-identity and character strengths*	*Modules 1–4*	*Module 4: Goal in life - Identify personal goals and work towards achieving these goals using game and stories.*
*Mental health – managing tension and our emotions*	*Modules 6, 9, 16*	*Module 6: Identifying emotions – use emoticon game to discuss different emotions – ways to manage anger and frustration.*
*Communication skills and self-esteem*	*Modules 5,7,8*	*Module 5: Communicating confidently –passive, confident and aggressive styles of communication – use of “I” messages and active listening skills.*
*Relationship skills and bouncing back – forgiveness and self-care*	*Module 10 and 12*	*Module 12: Accepting our differences social inclusion, how we regard ourselves and engage with others - group games, and discussions*
*Gender Transformative modules*	*Modules 11,14,15*	*Module 14 – Gender and Gender Based Violence –Gender as a cultural construct, sexual abuse/harassment, gender based violence, legal setting and community support structures*
*Planning skills and citizenship*	*Modules 13,17,18*	*Module 13 – I can create change - Collective decision making and planning for a joint project - each group develops and implements a small project for community benefit*

The current intervention is a co-adapted iteration of the intervention from earlier qualitative, quantitative and realist evaluations [26,27,30]. The studies demonstrated the effectiveness of the intervention to achieve the outcomes of resilience and gender equal attitudes [21,25,30]. The qualitative and quasi-experimental studies clearly established the impact of this group intervention [30] and facilitation by local women assured cultural acceptability and safety of the intervention [20–25]. The current iteration was built on strengths of other interventions modelled for impacting resilience and gender equal attitudes, especially in girls [8,9]. The key mechanisms identified in the realist evaluation [25] like fun activities triggering greater participation and mental health and peer facilitator role-modelling to trigger youth to re-evaluate their own gender attitudes and self-management skills [22]

The six facilitators were residents of informal urban communities who had completed high school comprising four women and two men, and five of six were aged under 30 years. They were supported by two coaches (implementation team members). The facilitators were trained in a series of four-hour workshops (total of nine, each covering two modules) and this included community organisation, group facilitation skills, and how to support young people with psycho-social disability.

## Fidelity, intervention and data collection quality

Data was collected by a team that was not involved in the implementation. Data collection tools, printed in Hindi, were filled by each participant. The data collection was done in groups of 10–15 participants, with two data collectors overseeing the process. Illiterate participants were supported by an additional person who read the questions aloud and wrote their responses. Two different people reviewed forms for completeness, and missing data were filled immediately. In a few instances, the missing data were filled by contacting the participant either in person or over phone. The digitisation of the assessment scores was done by an independent person, and digitally entered records were cross-checked for accuracy and cleaned, and gaps identified were addressed to ensure the quality.

Intervention quality was ensured through training sessions by coaches as described above. Coaching meetings for intervention group were initially held fortnightly and then weekly after module 10 (because facilitators described that more frequent review was needed). Coaching was conducted weekly throughout the intervention with the wait-control group. Facilitators recorded attendance, home visits, start and end time, presence of other members and self-scoring of facilitation. Facilitators home-visited and met those who missed a session to brief them on the contents they missed. Home activities were also monitored by the facilitators.

Fidelity checklists were filled by non-implementers and assessed adherence to intervention and participation by young people using paper-based registers that assessed module delivery and attendance as well as narrative text summaries by facilitators summarising what went well and challenges in each session. The fidelity of sessions scores was between 3.5 and 4.5 on a scale of 0.0–5.0 with a mean of 3.96.

### Psychometric tools used for data collection and analysis

To evaluate the primary outcome of youth resilience we used the following psychometric scales which have all been validated in India, in English and Hindi. The Hindi tools were translated with inputs from the participants and field teams, and this was to ensure that it can be used across different age groups.

- Connor-Davidson resilience scale (CD-RISC) – a 10-item scale to measure resilience with scores between 0 and 4, higher scores indicating higher resilience. This scale has a high internal validity and has been validated widely [33].
- Child Youth Resilience Measure (CYRM) – a 12-item scale to measure resources available to individuals that may bolster their resilience, it has a five-point scoring system, with higher scores indicating higher availability of resources for resilience. It has been used in India in young women where the internal validity for social and emotional assets was high [10,34].
- Schwarzer’s General Self-Efficacy Scale (SES), a 10-item scale to measure self-efficacy, with scores between 1 and 4 (higher scores indicating higher self-efficacy). This scale has an internal validity of Cronbach’s Alpha between 0.76 and 0.90 [32].

Because earlier studies of Nae Disha had identified gender relations and wellbeing as central mechanisms to trigger outcomes [27], we also examined the outcomes of gender equal attitudes and mental distress using the following two scales:

d.  Gender Equitable Measurement Scale (GEMS), an 8-item scale to measure the gender attitudes, responses are scored between 0 and 4 with a maximum score of 32. Higher scores indicate higher gender equality attitudes and is validated for use in India [35].
e.  General Health Questionnaire (GHQ12), a 12-item screening tool to measure psychological distress. GHQ was scored using Likert scoring where responses are scored on a four-point scale of 0–3 with a maximum score of 36. Higher scores indicate poorer mental health. This tool has been used in South Asians and young people [36] although it has not been formally validated among tribal young people in India.

Data was collected for all groups at T1 in March 2019 and at the completion of the all Nae Disha modules for the intervention group at T2 in September 2019. A third measure T3 in March 2020 measured changes in outcomes for 12 months post-intervention for the intervention group and immediately after the intervention in the wait-control group.

### Statistical analysis

To assess the effect of the intervention, a difference in mean scores of the participants for all five outcomes were assessed at baseline (T1) and at the end of the intervention (T2) we used a difference-in-difference (DiD) analysis [37]. The DID estimate was modelled using clustering and time-fixed effects, with the treatment occurring at the cluster and time levels [38]. In the adjusted model, the baseline sociodemographic data (age, caste, religion, participant education and literacy and parents’ education) were included. We therefore used the following (two-way fixed-effects) model:

yist = γs +γt + **z**istβ + Dstδ + εist

where yist is the outcome of person i in group s at time t, γs are cluster-fixed effects, γt are time-fixed effects, **z**ist are the covariates, β are the coefficients in the covariates; Dst is the (time-varying) treatment indicator, δ is the coefficient on the treatment indicator, and εist are the residual errors.

The wild cluster bootstrap inference approach was applied for inferential purposes to address the small number of clusters.

Given the testing of multiple outcomes, the Romano-Wolf multiple hypothesis correction was applied, but no changes in the significant p-values were observed when compared to the original model.

T3 measurements allowed us to analyse trends in outcomes for the two groups after the intervention was extended to the wait-control group, given that they had received the intervention between T2 and T3, using linear regression models. Coefficients and 95% confidence intervals were used to summarize the results and estimate inference in both the DiD and the linear regression analyses. All estimations were stratified for boys and girls.

## Results

We present participation with both a CONSORT diagram (Figure 2) and the socio-demographic profile of participants (Table 2) below.

**Table 2.** Sociodemographic profile of study participants.

Socio-demographic details	Boys		Girls	
	Intervention group (*n* = 109)	Wait-control group (*n* = 80)	Intervention group (*n* = 138)	Wait-control group (*n* = 114)
**Age in years**				
<13	3 (2.8)	4 (5.0)	1 (0.7)	4 (3.5)
13–15	68 (62.4)	64 (80.0)	78 (56.5)	64 (56.1)
16–18	32 (29.4)	10 (12.5)	51 (37.0)	40 (35.1)
>18	6 (5.5)	2 (2.5)	8 (5.8)	6 (5.3)
**Caste**				
General Caste	26 (23.8)	20 (25.0)	27 (19.6)	26 (22.8)
Backward Caste	33 (30.3)	22 (27.5)	43 (31.2)	31 (27.2)
Schedule Caste/tribal Other	22 (20.2) 28 (25.7)	14 (17.5) 24 (30.0)	34 (24.6) 34 (24.6)	17 (14.9) 40 (35.1)
**Religion**				
Hindu	51 (46.8)	47 (58.8)	68 (49.3)	71 (62.3)
Muslim	56 (51.4)	33 (41.3)	68 (49.3)	42 (36.8)
Others	2 (1.8)	0 (0.0)	2 (1.5)	1 (0.9)
**Participants – years of education completed**				
Primary	100 (91.7)	73 (91.3)	124 (89.8)	104 (91.2)
Secondary	9 (8.3)	7 (8.8)	14 (10.2)	10 (8.8)
**Mothers – years of education completed**				
Primary school (1–5 years)	46 (42.2)	33 (41.3)	55 (39.9)	46 (40.4)
6–10 years	63 (57.8)	47 (58.8)	83 (60.1)	68 (59.7)
**Fathers – years of education completed**				
Primary school (1–5 years)	69 (63.3)	43 (53.8)	72 (52.2)	66 (57.9)
6–10 years	40 (36.7)	37 (46.3)	66 (47.8)	48 (42.1)
**Literacy** Not able to read Able to Read	8 (7.3) 101 (92.7)	2 (2.5) 78 (97.5)	12 (8.7) 126 (91.3)	7 (6.1) 107 (93.9)

The majority of participants were aged 14–19 years. A higher prevalence of disadvantage in the intervention group was evident; for example, a higher % of participants were from Census defined ‘backward classes’ (boys − 66%, girls − 83%), while the wait-control group had a higher percentage of Hindus (boys − 93%, girls − 61.8%) and a higher percentage of General (advantaged) caste (boys − 29.4%). Other measures of disadvantage such as housing quality and years of education completed by parents were comparable across both groups (Table 2).

### Difference in differences (DiD) analysis

Table 3 presents the results of the DiD analysis [37] for all the outcomes in girls and boys in the intervention and control groups for both crude and adjusted models. The mean scores of the resilience measure, CD-RISC, improved significantly for intervention boys (DiD (adjusted) = 5.82; 95% CI: 1.57, 9.74) compared to wait-control boys, and also among intervention girls (DiD = 4.12; 95% CI: 2.14, 6.09) compared to wait-control girls. Results for other measures were not significant.

**Table 3.** Difference in difference (T2-T1) analysis for all the outcomes among intervention and control groups.

Measures	Intervention group	Wait control	DiD Crude (95% CI)	DiD Adjusted (95% CI)	Intervention group	Wait control	DiD Crude (95% CI)	DiD Adjusted (95% CI)
	Boys				Girls			
CYRM (resilience)								
Before	43.8	45.1			44.5	45.1		
After	46.2	44.2	3.33 (−0.40, 7.06)	3.24 (−0.57, 7.56)	44.9	46.5	−0.95 (−4.55, 2.65)	−0.78 (−4.56, 3.15)
CD-RISC (resilience)								
Before	21.3	23.1			22.6	24.7		
After	25.9	21.8	5.83 (2.10, 9.55)	5.82 (1.57, 9.74)	25.2	23.4	3.93 (1.80, 6.06)	4.12 (2.14, 6.09)
GSE (self efficacy)								
Before	28.8	27.9			27.8	29.4		
After	30.5	29.4	0.37 (−2.09, 2.83)	0.31 (−2.38, 2.94)	29.8	30.1	1.27 (−0.94, 3.48)	1.24 (−1.00, 3.53)
GEMS (gender equal attitudes)								
Before	20.61	20.78			22.2	22.4		
After	21.17	20.41	0.89 (−1.23, 3.01)	1.00 (−1.10, 3.18)	22.6	22.3	0.46 (−1.78, 2.70)	0.62 (−1.64, 3.01)
GHQ (mental health)								
Before	2.58	2.88			2.99	2.31		
After	2.20	3.00	−0.48 (−1.50, 0.54)	−0.57 (−1.53, 0.40)	2.97	2.34	−0.04 (−1.18, 1.10)	−0.10 (−1.39, 1.11)

Acronyms – DiD – Difference in difference, CI- Confidence Interval.

*P-values : CYRM – boys-0·1,girls-0.93; CD-RISC – boys–<0·01; girls-0·01; GSE - boys—0·98, girls-0·07; GEMS - boys-0·98, girls-0·25; GHQ - boys-0·37, girls-0·04.

### Changes in measures over time within the boys groups across T1,T2 and T3 (Table 4)

Boys in the intervention group showed statistically significant improvements in CD-RISC immediately and in both CD-RISC and CYRM (resilience) measures at six-months post-intervention, while wait-control boys also showed significantly improved resilience scores (CD-RISC, CYRM) and also improved scores in self-efficacy (GSE).

**Table 4.** Change in mean scores and CI intervals of outcomes at T1, T2 and T3 in intervention and wait-control groups for boys. Crude and adjusted models.

		Crude			Adjusted	
	T1	T2 β (95% CI)	T3 β (95% CI)	T2 β (95% CI)		T3 β (95% CI)
**CYRM**						
Intervention group	43.8	2.18 (−0.12, 4.48)	2·25 (−0.31, 4.81)	**2.35 (0.39, 4.31)**		**2.84 (−1.77, 7.44)**
Wait-control group	45.2	−0.74 (−3·29, 1·82)	1·76 (−0.81, 4.33)	−0·63 (−3.87, 2.60)		1·70 (−4.67, 8.07)
**CD-RISC**						
Intervention group	21.3	**4.49 (2.34, 6·64)**	**5.61 (3.43, 7.79)**	**4.78 (1.37, 8.18)**		**6.17 (0.94, 11.40)**
Wait-control group	22.9	−0.10 (−3.35, 1.14)	**3.64 (1.37, 5.90)**	−0.96 (−3.15, 1·23)		**3.66 (0.59, 6.74)**
**GSE**						
Intervention group	28.7	**1.69 (0.15, 3.25)**	**2.06 (0.49, 3.64)**	**1.77 (0.24, 3.29)**		2.33 (−0.06, 4.74)
Wait-control group	28.1	1.05 (−0.73, 2.84)	**3.53 (1.73, 5.32)**	1.27 (−0.54, 3.08)		**3.53 (0.41, 6.65)**
**GEMS**						
Intervention group	20.6	0.60 (−0.87, 2.07)	**1.90 (0.41, 3.39)**	0.74 (−1.13, 2.61)		1.84 (−0.38, 4.07)
Wait-control group	20.4	0.43 (−1.25, 2.11)	**2.66 (0.97, 4.35)**	0.21 (−1.97, 2.40)		2.50 (−0.87, 5.98)
**GHQ**						
Intervention group	2.5	−0.35 (−1.03, 0.32)	**−0.73 (−1.42, −0.05)**	−0.43 (−1·15, 0·28)		**-0.83 (−1.98, −0.31)**
Wait-control group	2·7	0.33 (−0.46, 1.11)	−0.54 (−1.33, 0.25)	0.35 (−0.49, 1.21)		−0.42 (−1.96, 1.11)

### Changes in measures over time within the girls groups across T1, T2 and T3 (Table 5)

Girls in the Intervention group at 6 months post-intervention had statistically significant improvements in their resilience measures (CYRM, CD-RISC) and self-efficacy (GSE) and these same measures were also observed to be statistically significant in girls from the wait-control group. The wait-control girls also had significantly improved improvements in their Gender Equal attitudes measure (GEMS).

**Table 5.** Change in mean scores and CI intervals of outcomes at T1, T2 and T3 in intervention and wait-control groups for girls. Crude and adjusted models.

		Crude		Adjusted	
	T1	T2 β (95% CI)	T3 β (95% CI)	T2β (95% CI)	T3 β (95% CI)
**CYRM**					
Intervention group	44.5	0.25 (−1.84, 2.33)	**4.23 (2.12, 6.33)**	0.48 (−2.54, 3.50)	**4.47 (1.32, 7.62)**
Wait-control group	45.5	0·86 (−1.15, 2.88)	**2.75 (0.74, 4.76)**	0.72 (−2.43, 3.87)	**3.08 (0.56, 5.59)**
**CD-RISC**					
Intervention group	22.5	**2.64 (0.61, 4.67)**	**4.11 (2.06, 6.16)**	**2.83 (1.39, 4.28)**	**4.33 (1.62, 7.04)**
Wait-control group	24.7	−1.26 (−3.41, 0.90)	2.88 (0.73, 5.03)	−1.47 (−3.21, 0.27)	**2.93 (0.26, 5.60)**
**GSE**					
Intervention group	27.9	**1.93 (0.46, 3.40)**	**3.98 (2.50, 5.46)**	**2.02 (0.45, 3.59)**	**4·07 (2.42, 5.73)**
Wait-control group	29.3	0.73 (−0.79, 2.24)	**1.57 (0.06, 3.08)**	0.69 (−1.38, 2.77)	**1.89 (0.75, 3.03)**
**GEMS**					
Intervention group	22.2	0.35 (−0.78, 1.49)	**1.37 (0.23, 2.52)**	0.45 (−1.41, 2.30)	1.51 (−0.90, 3.93)
Wait-control group	22.4	0.07 (−1.18, 1.31)	**1.99 (0.75, 3.23)**	0.07 (−1.82, 1.97)	**2.15 (0.85, 3.45)**
**GHQ**					
Intervention group	3.0	0.05 (−0.58, 0.68)	**−0.67 (−1.32, −0.04)**	−0.08 (−0.72, 0.56)	**−0.70 (−1.20, −0.19)**
Wait-control group	2.2	0.10 (−0.57, 0.77)	−0.22 (−0.88, 0.44)	0.05 (−1.06, 1.15)	−0.10 (−1.21, 1.01)

We also observed indirect benefits for young people that were not formally measured in this trial. These included the formation of new friends, increased freedom of movement and greater confidence speaking in class for young women. Parents reported that both boys and girls who had completed the intervention were more engaged in study and in completing home responsibilities.

## Discussion

To our knowledge, this is one among a handful of randomised controlled trials evaluating resilience and mental health interventions for young people living in a very low-income setting [12,19,21,22,38].

The improved resilience measures measured immediately post-intervention in the intervention group were further strengthened in the 6 months following completion of the active phase of the intervention, a finding observed across all three of five measures for girls, and in two of five measures for boys. This differs from the pilot intervention of Nae Disha, where improved youth resilience outcomes decayed 6 months post-intervention [30]. Lay facilitators proposed that the ongoing relationship maintained with young people may have supported this outcome, consistent with the findings of a resilience intervention among young women in Bihar which showed gains in resilience were sustained with continuous relational engagement and no additional component of intervention [39].

It seems likely that similar mechanisms to those in the realist evaluation of a Nae Disha implementation conducted 3 years earlier might operate among young people in this study: namely, improved social confidence and peer friendships, leading to greater autonomy, and freedom of movement (for girls) as well as increased social inclusion (for boys) [27]; however, there was no improvement in the outcome of self-efficacy unlike the earlier studies. Several studies published after the completion of this trial have showed a wide scope of benefits for young people (and particularly young women) who participate in groups [22], for example, a study examining a similar intervention to ND3 in Zambia, showed modest improvements in financial literacy, self-efficacy and sexual and reproductive behaviour but did not impact some bigger picture health determinants such as gender equity norms and educational outcomes, an intervention conducted in Pakistan concluded that a community-based intervention is more acceptable and improves resilience and mental health [22,38].

On the resilience scales, the magnitude of impacts was larger in girls compared to boys; however, this difference is not statistically significant. One reason for this might be that girls showed higher attendance and further, the intervention format of group meetings, and discussions may be better suited to young women in this setting where young women are typically more sedentary with less freedom of movement [40] and perhaps are more skilled at social conversation [27,41,42]. It is likely that the group platform supported young women to form new peer friendships, a mechanism supporting outcomes, in another group intervention conducted among disadvantaged young women in India [23]. Poorer attendance, especially for young men, also meant a lower dosage of intervention and this could have potentially reduced the statistical significance in the results, given the limited sample, age-specific analysis would produce unstable estimates.

Interestingly, the wait-control group benefited more at 3 months after intervention than the intervention group. We propose a key reason for this is that facilitators delivered a more high-quality intervention (greater fidelity) and were more confident in delivering the Nae Disha intervention the second time around and were also more actively coached, following learnings from the first group [43].

Though the improvements in our primary outcome, resilience, was statistically significant, the secondary outcomes in the DiD analysis were not. This could be for two reasons: first, the rigid RCT recruitment process led to high numbers of youth who completed baseline measures but then did not participate in the intervention. This differed from the recruitment process we used in earlier non-trial settings, which allowed a period of several weeks of flexible attendance and try-outs prior to implementation of the intervention. Second, the rigid intervention required by this trial methodology also reduced the opportunity for bespoke variations (such as playing volleyball before the group meetings) unlike earlier iterations.

However, it should be noted that the changes in mean scores for the control group were statistically significant for several secondary outcomes in both intervention and wait-control groups at T3. We note that while post-intervention measures were not collected from the wait-control group, their mean scores after the intervention showed significantly better outcomes than those of the intervention group.

Like another group intervention study in Zambia, this study did not find meaningful improvements in the gender equal attitudes measure in young men, although there were significant improvements in the pre-post measures among the wait-control girls group. Recognising the relative disadvantage of our sample, it is possible that this study could not adequately address the many diverse factors that support unequal gender attitudes in hegemonic masculinity in India. Any intervention addressing gender relations is likely to require time, engagement with entire families and systemic changes to gender relations in communities, governance and educational institutions [9,18]. We note the efficacy of a ‘gender-equality conversations’ intervention in Indian high schools in Haryana which generated changes that lasted over 2 years, as a possible approach to strengthen changes in gender equal attitudes in the future implementation [19]. It seems likely that an ongoing (post-intervention) relationship of young people with others, who challenge typical gender relations as key to gender attitude change as found in other youth interventions in India [9,23,27].

## Implications

This randomised controlled trial suggests that the ND3 intervention was moderately effective at improving resilience for both young men and women but did not improve mental health and gender equal attitudes overall among these highly disadvantaged young people in urban North India. The attendance and outcomes suggest it is more acceptable, feasible and effective to improve resilience for young women. These findings add to previous evaluations to support the value of this 18-week intervention for use in community or school groups [25–27,43].

The study adds key evidence to support the value of psychosocial group interventions for young people to improve resilience outcomes. We propose a need for further research using a larger sample size and an intervention design that accommodates some responsiveness to diverse small groups, as well as a longer time period of follow-up. We also believe it would be valuable to evaluate whether this intervention can contribute to suicide prevention.

## Methodological considerations

There were important strengths in the study design: First, it was conducted in a low-income ‘real-life’ setting and groups were facilitated by community members who understood the local contexts well, thus likely delivering the intervention in ways that were relevant and acceptable. The implementation was relatively low-cost because it was facilitated by local community members who received modest remuneration and used local resources (such as holding meetings in local houses) which increased generalisability and minimised costs. The assessments used validated psychometric measures which added to the strength and generalisability of this study. The cluster randomised methodology formed groups of young people who lived near each other, which imitates likely implementation in a community and makes it more generalisable than a trial randomised at individual level.

The intervention had been developed and further adapted for implementation in this setting and provided culturally relevant examples and content, as well as a process of implementation that was culturally acceptable while challenging gender norms linked to patriarchy. Other studies have shown that cultural adaptation can enhance the efficacy of psychological interventions [44].

There are some important limitations to this study. First, attendance was lower than in our previous iterations of Nae Disha which reduced possibilities of finding an association between changes in scores and participation. The attendance might have been negatively impacted by the controlled trial methodology which did not allow a more organic group membership. In earlier iterations of Nae Disha there was typically a 3- to 4-week period where young people could join or leave the group before settling into a regular group. In this trial format, we had to limit the joining of young people after the initial recruitment. Second, the wide age range used was pragmatic (we included young people of a wide age range who requested to participate from the selected lanes) in this setting with no other support for mental health, however it might be that the intervention was less relevant for the youngest and oldest participants. However, results could not be disaggregated for these age groups due to small numbers. Third, the implementation of the Nae Disha intervention among the wait-control group before they completed the T3 measure reduced the ability to assess the long-term impact of the intervention as a control to the intervention group and can only suggest an aggregate trend but cannot confirm the program impact. Fourth, in spite of randomisation, the wait-control group had relatively more advantaged participants. We adjusted by conducting an analysis with a balanced sample. The asymmetries in the sample do not detract from the results of this study which show some benefits for both groups. Fifth, as only two-thirds of the original baseline sample were ultimately recruited and analysed, the design and test combination might have been underpowered for detecting hypothetical effect sizes of interest. We also acknowledge that attrition bias might have influenced the statistical power of the study and the balance of confounders between the groups. Finally, the lower improvement in the CYRM measure compared to the CD-RISC measure suggests variations in the validity of these two measures which merits further review in research studies ahead.

## Conclusions

This community-based trial suggests that the ND3 psychosocial group intervention moderately improved resilience and self-efficacy for both young men and women in marginalised urban communities in North India although it did not yield significant improvements in mental health or gender equal attitudes. As a low-cost intervention that has been shown to be relevant, acceptable and feasible, it can be implemented by lay community-based facilitators who are supportively coached. While this study was set in low-income urban communities of North India, it is likely that this youth resilience intervention may also be effective in other South Asian settings and beyond.
